# Biocompatibility Assessment of Conducting PANI/Chitosan Nanofibers for Wound Healing Applications

**DOI:** 10.3390/polym9120687

**Published:** 2017-12-08

**Authors:** Panagiota Moutsatsou, Karen Coopman, Stella Georgiadou

**Affiliations:** Department of Chemical Engineering, Loughborough University, Loughborough LE11 3TU, Leicestershire, UK; P.Moutsatsou@lboro.ac.uk (P.M.); K.Coopman@lboro.ac.uk (K.C.)

**Keywords:** polyaniline, chitosan, electrospun nanofibres, wound dressing, conductive fibres

## Abstract

As electroactive polymers have recently presented potential in applications in the tissue engineering and biomedical field, this study is aiming at the fabrication of composite nanofibrous membranes containing conducting polyaniline and at the evaluation of their biocompatibility. For that purpose, conducting polyaniline–chitosan (PANI/CS) defect free nanofibres of different ratios (1:3; 3:5 and 1:1) were produced with the electrospinning method. They were characterized as for their morphology, hydrophilicity and electrical conductivity. The membranes were then evaluated for their cellular biocompatibility in terms of cell attachment, morphology and cell proliferation. The effect of the PANI content on the membrane properties is discussed. Increase in PANI content resulted in membranes with higher hydrophobicity and higher electrical conductivity. It was found that none of the membranes showed any toxic effects on osteoblasts and fibroblasts, and that they all supported cell attachment and growth, even to a greater extent than tissue culture plastic. The membrane with the PANI/CS ratio 1:3 supports better cell attachment and proliferation for both cell lines due to a synergistic effect of hydrophilicity retention due to the high chitosan content and the conductivity that PANI introduced to the membrane.

## 1. Introduction

Electrical stimuli have been shown to have a positive impact on *in vitro* cell cultures of electroactive tissues, promoting cell adhesion, alignment, regulating and modulating cell differentiation, migration, protein secretion and DNA synthesis [1,2]. The response of fibroblasts and osteoblasts to electric stimulation with a view to enhancing wound and bone fracture healing has only recently been assessed *in vitro* [3,4], *in vivo* [5,6], and in some clinical studies [7,8], indicating the potential advantages of electrical stimulation of these specific tissues, even though they are not generally classified as electroactive (when compared to muscle and nerve for example). These *in vitro* and *in vivo* studies refer to electrical stimulation of the tissue usually through the use of a hydrogel or an electroactive flat membrane produced by film casting, whereas, in clinical studies, the electrical stimulation is achieved with direct attachment of electrodes on the wounded area or with the use of aluminium foil. However, the latter procedure may increase the risk for contamination of the wound, being disruptive to the healing process [9]. A porous, conducting wound dressing would be ideal to apply electric current at required intervals, without the need to expose the wound. A wound dressing that could hold pre-seeded cells for the introduction of cytokines, collagen and growth factors to the wound bed [10], or the encapsulation and controlled release of bioactive molecules from the wound dressing would further assist and accelerate the healing process. A nanofibrous structure would add value to such wound patch, by increasing the surface to volume ratio, and thus the quantity of the loaded molecules. Furthermore, the need for nanofibrous structures has been highlighted by several researchers who showed how the nanofibrous topography apart from the obvious advantage of the large surface to volume ratio, also seems to provide significant advantages for cell culture applications as they have been found to induce enhanced cell activity when compared to flat surfaces composed of the same materials. For example, Chu et al. showed how hepatocytes attach better on chitosan nanofibres, rather than on chitosan flat film, also exhibiting higher urea synthesis, albumin secretion and cytochrome P450 activity. Furthermore, when nanofibres are compared to microfibres, they have been found to outperform those, in terms of cell activity (proliferation, excretion of extracellular matrix, maintaining their initial morphology and differentiation state) [11,12]. From all the above, it becomes obvious that a nanofibrous structure made of electroactive polymer would be very beneficial for the enhancement of new skin tissue formation. Electrospinning has emerged as a very robust method for the production of nanofibrous structures from a large variety of polymers, combining efficiency, versatility and low cost. It is a widely used technique, firstly patented by Formhals in 1934, but which has been continuously evolving ever since (e.g., co-axial [13], upward [14], needle-less [14], alternating current [15,16], wet electrospinning [17], etc.), broadening and tailoring even further the properties of the produced nanofiber morphologies, depending on the desired application. 

Polyaniline (PANI) is a polymer whose conjugative structure allows the mobility of electrons on its chain, rendering it electrically conducting. Preliminary studies on polyaniline are showing that it could be a potential medium for the transfer of electrical stimuli to tissues, enhancing the control over differentiation and orientation of specific types of cells such as skeletal muscle, nerve and cardiac tissue [2,18]. Chitosan is the *N*-deacetylated derivative of chitin, a polymer encountered in nature which is biocompatible, biodegradable and has been found to exhibit antibacterial, antifungal, mucoadhesive, immunological, hemostatic [19] and wound healing properties, as well as to promote cell adhesion and proliferation. It is processable in slightly acidic aqueous solutions and can be easily cast as a film, form hydrogels or be electrospun with the presence of other polymers [20,21,22]. Polyaniline on the other hand, has shown more contradictory results in terms of biocompatibility; preliminary *in vitro* and *in vivo* biocompatibility studies have not been conclusive on this matter. For example, Zhao et al. [23], found that the electroactive quaternized chitosan hydrogels containing polyaniline could significantly enhance the proliferation of C2C12 myoblasts compared to the pure quaternized chitosan hydrogel. Similarly, incorporation of PANI in polyethylene glycol based hydrogels has been shown to promote the cell response of PC12 (pheochromocytoma of the rat adrenal medulla cells) and hMSC’s (human mesenchymal stem cells) as a result of the increase in conductivity and water retention that PANI caused [24]. It should be noted that for wound healing purposes, the use of nanofibres as opposed to hydrogels can be proved advantageous, as although the first are known to ensure wound hydration [25] they are outperformed by 2-D nanofibrous meshes as for hemostasis, cell respiration, and gas permeation when implanted onto open wounds [26]. Additionally, water accumulation due to the hydrogel can cause maceration and bacterial proliferation [27]. 

Wu et al. studied the proliferation and morphology of L929 fibroblast cells on electrospun polycaprolactone (PCL) and PCL-PANI fibres with different ratios of contained PANI at 1%, 5%, 10% and 20% *w*/*w* and they concluded that no difference was observed with respect to growth rate and morphology when compared to Tissue Culture Plastic (TCP), suggesting that PANI does not have any cytotoxic effect on the cells. The PCL-PANI 20% gave slightly higher number of cells at the end of the culture time (4th day) [28]. However, in most of these studies, the amount of reproducibly incorporated PANI in the structure does not exceed 5% *w*/*w*, which is a limitation caused by PANI’s lack of processability. This is important, as it is expected that incorporation of higher PANI contents, would increase percolation in the nanofibrous structure and provide higher conductivity for the induction of electrical stimuli and improved electrical properties for controlled release applications and in general better control over the electrical properties of the produced membrane. When higher concentrations of conducting polymer are used, there is the possibility of an adverse effect regarding biocompatibility as indicated in the study of Ma et al. [29], for example. In this study, cast film of chitosan grafted with electroactive aniline tetramer was found to enhance cell proliferation of chondrocytes and C2C12 myoblasts as compared to the pure chitosan one, but high content of grafted aniline tetramer had an adverse effect in terms of cell viability. It is possible though that the adverse effect caused by the use of aniline tetramer instead of polyaniline, as it has been shown (Zhang et al. [30]) that PANI oligomers may show cytotoxicity towards certain types of cells such as the NIH-3T3 fibroblasts that were used in that study, even though in a different study the same type of cells was proven to be positively affected by subjection to various current flows [31]. There have also been cases where *in vivo* implantation of PANI scaffolds has resulted in inflammation and poor biocompatibility [32], although this could be potentially attributed to the remaining aniline monomers and oligomers, but also because of remaining solvents and acids used during the polymerization or the processing of PANI and not because of the polymer itself [2,33]. 

The matter of PANI biocompatibility is still open for investigation within the scientific community and therefore there is a need is to determine firstly if the presence of PANI compromises cell viability and at a second stage, whether the incorporation of conducting PANI in polymeric scaffolds and wound dressings can be proven beneficial to cell attachment and proliferation. If this is true, encapsulation and release of bioactive molecules in a pulsatile ON/OFF way will be possible with electrical stimulation of the scaffold through electrodes, causing the fibers to release the desired substance via small cracks created on the nanofiber surface caused by actuation as explained by Abidian et al. [34]. The potential for using external electric field on polymeric biodegradable scaffolds to electrically guide cell differentiation and direction of growth will also be reached. This study is therefore focusing on answering the question of whether the incorporation of high amounts of PANI in a biocompatible substrate, affects biocompatibility in terms of cell attachment, growth and spreading. To avoid remaining aniline monomers that could tamper with the results, commercial PANI was used in this study, and all cell biocompatibility assays were performed after thorough washings of the PANI containing membranes. This is determined by means of successfully electrospinning novel blend PANI/Chitosan (PANI/CS) nanofibres, and more importantly at high PANI ratio (50:50), which could find applications in other fields too, where engineering nanofibres with high amounts of conducting polymer is of fundamental importance. This is the first time that a porous electroactive nanofibrous membrane is examined for its potential use in wound dressing applications, with promising results. 

The aim of this study is therefore to produce defect free nanofibres from blends consisting of conducting PANI and biodegradable chitosan, in an attempt to combine the positive effects of both, then to evaluate their biocompatibility and electrical properties as well as to assess whether the incorporation of PANI in the fibres is beneficial to cells. To address this purpose, some important morphological and physical properties of the nanofibrous blend mats will be measured such as fiber diameter, mat conductivity, hydrophilicity/hydrophobicity and most importantly the biological *in vitro* compatibility of these electrospun membranes will be assessed with human osteoblast and fibroblast cells using standard viability/cytotoxicity tests. 

## 2. Materials and Methods

### 2.1. Electrospinning of Chitosan-Polyaniline Blends

Trfiluoroacetic acid (TFA), dichloromethane (DCM) and chitosan of molecular weight (*M*_w_) 600,000–800,000 and 90% degree of deacetylation, which were purchased from Acros Organics (ThermoFisher Scientific Inc., Loughborough, UK); as well as CSA ((1R)-10-camphor-sulphonic acid) and polyaniline of Mw 50,000 which were purchased from Sigma Aldrich Inc. (Gillingham, Dorset, UK)were used to prepare solutions of pure chitosan (control) as well as blend solutions of doped PANI and chitosan (PANI/CS), at ratios 1:3, 3:5 and 1:1 and at a constant concentration of chitosan at 5% *w*/*v*. The blends were fed through a plastic syringe to the needle tip (20 G diameter) and were electrospun under different voltages, produced by a high voltage source (Glassman High Voltage Inc., Hampshire, UK). The nanofibres were collected on a flat grounded collector covered with aluminium foil. The needle tip to collector distance was fixed at 16 cm at a horizontal orientation. The flow rate of the solutions was controlled by a syringe pump (Harvard Apparatus, Cambridge, UK). The humidity in the electrospinning chamber was set at 30% RH by a constant dry air flow and monitored using a temperature and humidity meter ST-321. The temperature was monitored by the same device. The morphology of the electrospun membranes was then examined by using a Carl Zeiss (Leo) Scanning Electron Microscope (Model 1530VP, Zeiss, Oberkochen, Germany) and the average diameter of the fibres was measured using the AxioVision software (LE 4.8.2.0, Zeiss, Oberkochen, Germany).

### 2.2. Water-Proofing of Electrospun Membranes

As the chitosan salt is soluble in water, the as spun membranes disintegrate when immersed in aqueous media for the cell culture. For that reason, a post spinning process was required to render the membranes insoluble. In the present study, a neutralization process was used to deprotonate the chitosan molecule and render it insoluble in water. Three saturated salt solutions that are suggested in the literature [19,20,35], were compared as for their efficiency to “waterproof” the electrospun membranes containing chitosan and polyaniline. The electrospun membranes were not removed from the aluminium foil after electrospinning and dipped as such in saturated water solutions of Na_2_CO_3_ or NaOH for 3 h or in a 90:10 methanol:water NaOH solution for 10 min, as suggested in the abovementioned studies. Soon after treatment, they were detached from the aluminium foil and could be easily handled. After that, they were thoroughly washed and immersed in phosphate buffered saline (PBS) of pH 7.4 (ThermoFisher Scientific Inc., Loughborough, UK), at 37 °C for several days and were tested as for their morphology under a scanning electron microscope in specified time intervals to monitor any changes on the nanofibrous structure such as fusion of fibres, increase in diameter size, membrane disintegration in the aqueous media during the course of that time period. 

### 2.3. Contact Angle Measurement

Cells are susceptible to changes of the hydrophobicity/hydrophilicity of the culture surface; therefore the contact angle of the electrospun membranes was measured using the sessile drop method, with a contact angle measuring and drop shape analysis system (DataPhysics OCA, DataPhysics Instruments GmbH, Filderstadt, Germany) and the use of a water droplet of water to calculate the contact angle.

### 2.4. Electrical Resistivity 

Measurements of the membranes conductivity were performed to confirm homogeneous and continuous distribution of the PANI in the nanofibrous membrane and to examine the effect of the PANI content in the membrane as well as changes occurring during the neutralization step. The electrical resistivity of the electrospun membranes was measured using the four-probe technique as per ASTM F76. A Keithley 6220 DC current source was used to generate a direct current (DC) current I at the range of 20 μA to 100 mA, and the voltage through the sample was measured in four different directions with a Keithley 2000 DMM voltameter (Tektronix Ltd., Berkshire, UK). Each sample was measured three times and average values are reported.

### 2.5. Cell Culture

Two types of human cells were used for cell cultures to assess or evaluate cell viability/behaviour, osteoblasts (hOST-T85 cell line from eCACC) and fibroblasts (Neonatal foreskin human dermal fibroblast cells from Intercytex). Human osteoblast cells were grown in Minimum Essential Medium (MEM), supplemented with 10% *v*/*v* fetal bovine serum (Invitrogen), 1% *v*/*v* non-essential amino-acids (NEAA) and 2 mM l-glutamine and incubated at 37 °C/5% CO_2_. Human dermal fibroblasts were grown in Dulbecco’s modified Eagle’s medium (DMEM; Gibco), supplemented with 10% *v*/*v* fetal bovine serum (Invitrogen), and 2 mM l-glutamine. Both cell types were thawed and grown for two days in T-175 tissue culture flasks before being seeded on the electrospun membranes. They were then thrypsinised for 5 min to detach from the flasks, centrifuged and re-suspended in growth medium and finally counted with a nucleocounter NC-3000 using a viability and cell count assay Via1-Cassette™ by Cell Tech Group Ltd. (Berkshire Slough, UK). For the cell culture, the electrospun samples were cut into small round pieces (*d* = 2 cm), were washed twice with sterile PBS solution and sterilized under UV light for 1 h on each side. They were then placed in 6-well Ultra low attachment tissue culture well plates and secured at the bottom of each well with cell culture filters from which the bottom mesh has been previously removed. The electrospun membranes were soaked in cell growth medium overnight prior to seeding the cells previously grown in T-175 tissue culture flasks. 

### 2.6. Cell Attachment and Viability Assay

To evaluate the cell viability, a LIVE/DEAD^®^ Viability/Cytotoxicity Kit (ThermoFischer Scientific, Loughborough, UK) was used 3 days after cell seeding. The growth medium was removed and the seeded membranes were given a gentle PBS wash. A PBS solution containing 0.2% of ethidium homodimer dye (dead staining) and 0.05% calcium dye (live staining) was then added in the wells and left to incubate for 40 min. The samples were visualized using a Leica DMRX fluorescence microscope (Leica Microsystems Ltd., Milton Keynes, UK) equipped with the appropriate fluorescence filters. Digital images were acquired using a DS-Qi1Mc Nikon digital camera (Nikon Instruments Europe BV, Amsterdam, The Netherlands).

### 2.7. Cell Proliferation Assay

Live human osteoblasts of passage number 25 and fibroblasts of passage number 5 were seeded at a density of 4 × 10^4^ per well, in duplicates on the electrospun membranes and on tissue culture plastic surface (as a positive control). Additional 3 mL of the corresponding cell culture medium was added in each well. The seeded membranes were incubated at 37 °C/5% CO_2_. Cell attachment and proliferation were measured with a continual fluorescence assay, using AlamarBlue^TM^ (AB) (ThermoFisher Scientific, Loughborough, UK). Following overnight attachment in growth medium in a tissue culture incubator, supernatants were removed and 3 mL fresh complete medium containing 10% (*v*/*v*) AB was added into each well. After another 5 h incubation, triplicate 200 mL aliquots of the AB containing medium was removed from each well and put in a black 96-well microtiter plate for fluorescence measurement. The fluorescence was read at emission and excitation wavelengths 530 and 590 nm, respectively, using a FLUOstar Omega Microplate Reader (BMG LabTech, LabTech, Aylesbury, UK). Subsequently, fresh medium without AB was replaced in the wells. For continual assessment of cell proliferation, the AB assay was performed every other day on the same cell population for up to 6 days.

### 2.8. Morphological Assessment

For morphological assessment, samples in glass slides and tissue culture plastic were washed with PBS and then visualized on a Nikon eclipse Ti microscope (Nikon Instruments Europe BV, Amsterdam, The Netherlands). Digital images were acquired using a DSFi1 digital camera (Nikon Instruments Europe BV, Amsterdam, The Netherlands). As the morphology of the cells cultured on various membranes could not be assessed with normal contrast phase microscope (due to membrane opacity), the samples were prepared for scanning electron microscopy. They were fixed with 2.5% glutaraldehyde in PBS for 24 h at 4 °C, washed with PBS, and subsequently dehydrated in 15%, 30%, 50%, 70%, 85%, 95%, and 100% (twice) graded ethanol for 10 min each. They were left to dry in desiccator overnight and they were then sputter coated with Au/Pd for 60 s, and visualized with a scanning electron microscope (Carl Zeiss (Leo)—Model 1530VP).

## 3. Results and Discussion

### 3.1. Fabrication and Neutralization of Chitosan-Polyaniline Electrospun Membranes

Until recently, the electrospinning of pure chitosan solutions has proven challenging. Chitosan aqueous acidic solutions are very viscous with high surface tension which hinders the production of uniform nanofibrous membranes. In addition to that, the protonation of its free amino groups that takes place when chitosan is dissolved in acidic media, renders it a polyelectrolyte. Therefore, the repulsive forces between ionic groups within the polymer backbone that arise due to the application of a high electric field during electrospinning restrict the formation of continuous fibres and hinder the electrospinning process [20]. Pure chitosan nanofibres have only recently been successfully produced from trifluoroacetic acid solutions (TFA). Hasegawa et al. studied the dissolution of chitosan in TFA and concluded that dissolution occurs due to the formation of amine salts at the amino groups of C2 (2nd atom of carbon on the chitosan molecule) with TFA and also noted that no trifluoroacetylation occurs at the hydroxyl groups of chitosan [36]. TFA has also low boiling point and low surface tension which renders it a suitable solvent for electrospinning. It has also been shown that addition of a small proportion of dichloromethane (DCM) facilitates even further the electrospinning of chitosan as it further reduces the boiling point of the solvent system and also reduces the extremely strong charge density originated by the TFA [37]. Recently, it has been used as a solvent for polyaniline as well, in a thin film production process using the drop casting technique but the electrospinning of polyaniline or polyaniline/chitosan blends has never been tried before [38]. 

For these reasons, a TFA:DCM (80:20) solvent system was selected in the present study to produce polyaniline-chitosan blend solutions and electrospin them into nanofibrous structures. 

Based on a preliminary study regarding the electrospinnability of polyaniline-chitosan which is not presented here, the blend solutions were electrospun using the parameters summarized in Table 1. The resulting fibre diameters were measured (*n* = 150 for each membrane) with the aid of the AxioVision software. 

Given that the concentration of chitosan was kept at 5% *w*/*v* to ensure electrospinnability of the solutions, by increasing the ratio of PANI in the blends, the total polymer concentration was increased as well so that, in the blend with ratio 1:1, the total polymer concentration is 10% *w*/*v*. This made it impossible to keep the electrospinning parameters the same for all the electrospun blends. Therefore, at high PANI contents, the flow rate had to be lowered from 1 mL/h to 0.3 mL/h, and higher electric field needed to be applied in order to surpass the higher surface tension of the more concentrated solutions. Even with the decrease in flow rate and increase of conductivity, larger diameters are observed as the PANI content increases which is in contrast with what is usually stated in the literature where higher solution conductivity and decreased flow rate is usually associated with increased whipping, leading to thinner diameters. This increase in average fibre diameter can be explained firstly by the higher total polymer concentrations, and higher viscosity, as described above and secondly by the depletion of the tangential electric field occurring during electrospinning of highly conductive solutions as explained by other studies [39,40], which seem to prevail in this case.

**Figure 1:**
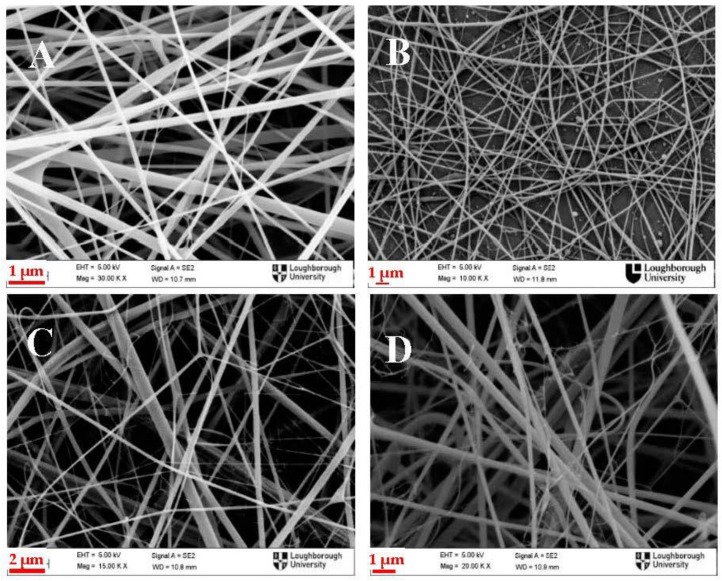
SEM images of: (**A**) Pure Chitosan; (**B**) Polyaniline/Chitosan (PANI/CS) 1:3; (**C**) PANI/CS 3:5; and (**D**) PANI/CS 1:1 electrospun membranes. Images are representative of three membranes prepared for each condition.

Additionally, increased solution conductivity due to higher PANI content in the blend required lower environmental humidity to make electrospinning feasible. This is because higher charge mobility within the jet facilitates charge exchange between the surrounding water vapours and the jet, resulting in removal of charges from the jet and therefore requiring higher voltage to counteract this phenomenon. In a previous study the combined effect of humidity, applied voltage and flow rate on the electrospinning of conducting polymer solutions had been explained thoroughly [40]. Briefly, as the flow rate is being decreased, and higher voltages are used, the process becomes more unstable, resulting in higher charge mobility and charge density on the whipping jet, and thus causing it to split into smaller subjets. This jet splitting phenomenon is the cause of higher standard deviation (SD) values regarding the nanofibre diameter (Table 1). This can be visually observed in Figure 1, where the membranes with the higher PANI content (C&D), presenting a lot of thinner and short fibres, characteristic of the jet splitting [41].

Table 1 shows that decrease in flow rate results in nanofibres with larger diameter distribution range. Although the standard deviation is usually used as a marker of membrane uniformity, the relative standard deviation was preferred over the standard deviation for this purpose, as a more appropriate statistical magnitude, given that it is independent of the measuring unit.

However, for the solutions which were electrospun at the same flow rate and applied voltage, pure chitosan & 1:3 and 3:5 & 1:1, a decrease of the RSD is observed from 53% to 38% and from 95% to 78% respectively which shows that the increasing PANI content has a positive effect in terms of membrane uniformity. Although jet splitting usually occurs in more concentrated and viscous solutions, and when higher electric field is used [41], in this case it seems that the higher concentration due to increased PANI content, as well as the increased charge per unit area due to the conducting PANI, have the opposite effect. This can be explained by taking into consideration that the high mobility charges introduced by the dopant acid, in this case CSA, offer higher charge mobility on the jet, balancing off the high charge density at the surface of the jet, which are generally the cause of the jet branching. The polarity of the chitosan itself must be also taken into account here. Chitosan is a positively charged molecule and it has been reported that when electrospun under positive high voltage (like in the present study) multiple jets are formed, instead of one, indicating process instability [42]. The addition of PANI in this case here (Figure 1B in comparison to Figure 1A, and Figure 1D in comparison to Figure 1C, which were electrospun under the same conditions but had higher PANI contents), seems to be counteracting this phenomenon, probably due to the dopant acid, in this case CSA, as is explained above, resulting in more uniform fibres. 

**Figure 2:**
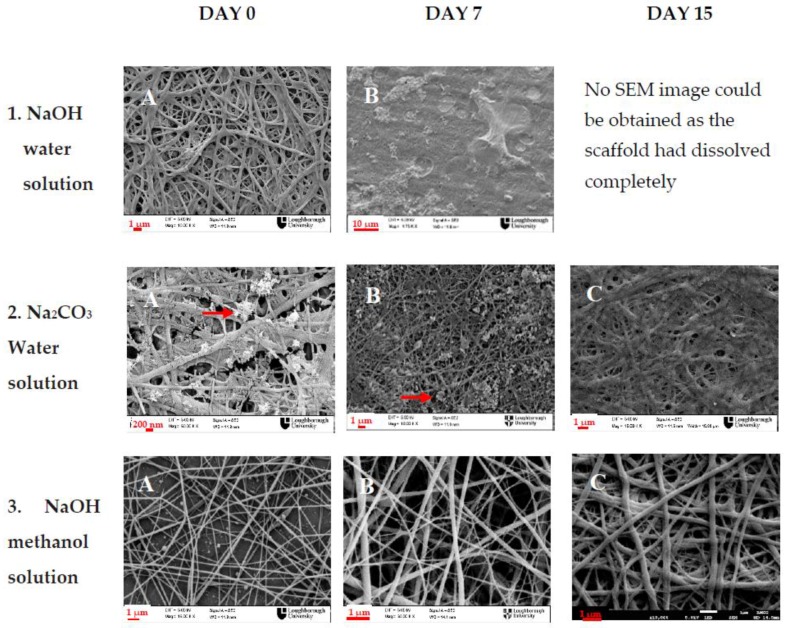
Electrospun chitosan containing membranes after neutralization and immersion in Phosphate Buffered Saline (PBS) after 7 and 15 days.

For the waterproofing step of chitosan containing mats, three methods were tested as for their efficiency to neutralize the electrospun membranes. The PANI/CS membranes were dipped in 5 M NaOH water solution, 5 M NaOH methanol/water solution (90:10) or 5 M NaCO_3_ solution. The morphology of the nanofibrous membranes was examined after 0, 7 and 15 days of immersion in PBS. The results are summarized in Figure 2.

A pH 7.4 PBS solution was considered to be suitable and convenient for testing the stability of the neutralized membranes; the PBS solution supports the osmotic balance of cells and has the same pH as the cell culture medium. As the *in vitro* culture time of both osteoblasts and fibroblasts usually does not exceed 14 days, the time frame of 15 days was also chosen on this basis. It is also in accordance with the time frame for a potential wound healing application [43]. Moreover, potential loss of structure is expected to happen because of the chitosan rather than the polyaniline since it is the more biodegradable of the two. The main mechanism of chitosan degradation in cell culture conditions would be by hydrolysis, which is mainly dependent on pH and temperature. Therefore, immersion of the neutralized membranes in PBS solution of pH 7.4, at 37 °C over a period time of 15 days is expected to be sufficient to draw conclusions on membrane stability. The membranes were however kept even after that 15-day period to check on the long term degradation rate. Day 0 is defined as the day that treatment was carried out (SEM images were taken immediately after treatment) and the morphological differences observed are due to the different treatment that each membrane received. As shown in Figure 2, the NaOH aqueous solution, although initially it seemed successful in water proofing the electrospun membrane, as the membrane retained its nanofibrous structure after immersion in the neutralizing water based solution (Figure 2(1A)), when it was left one week in PBS solution, the nanofibrous structure was completely lost (Figure 2(1B)). After 15 days, all the nanofibrous membrane has dissolved in the PBS. The neutralization with saturated Na_2_CO_3_ solution worked better, as the membranes were successfully waterproofed and the nanofibrous structure was retained even after two weeks in PBS (Figure 2(2B,C)); however, the excess salt precipitated (shown by arrows on Figure 2(2A,B)) on the nanofibres and although it was thoroughly washed, Na_2_CO_3_ remains were still present on the membrane after one week of immersion in PBS. Based on these results, the NaOH aqueous methanol solution, being the faster neutralization method (10 min) seemed to maintain best the nanofibrous structure of the membranes, and when checked even after 30 days of immersion in PBS, the nanofibrous morphology was still intact. No change in nanofibre diameter was observed after the neutralization process. It has to be noted, that, after immersion in alkaline NaOH methanol solution, partial dedoping of PANI occurs. This was observed visually as a gradual change of colour of the electrospun membranes from deep green, to blue and it was further investigated with conductivity measurement of the membranes, which will be analysed in the section to follow.

Different crosslinking methods of chitosan have been proposed in the literature, e.g., with glutaraldehyde [20] which could by-pass this problem. However, in this study, where the investigation of potential toxicity was the main focus point, cross-linking with glutaraldehyde would be compromising as it has been found to be toxic to biological tissues. There is also the possibility that, different dopant acids for the polyaniline could be good candidates for sustaining the electrical conductivity of PANI at high pH values [44]. However, it is generally accepted that for biomedical applications, polyaniline will inevitably undergo partial dedoping under physiological conditions where the pH is around 7.4. However, for wound healing applications, this is not restrictive, given that skin exhibits acidic pH values (<5) [45], which renders polyaniline a very good candidate for this kind of applications. Here, the cell culture was performed in normal pH conditions, mostly to prove the biocompatibility of the blend membranes as a first step rather than investigate the full potential of its conducting properties.

### 3.2. Characterization of Neutralized Electrospun Membranes

The membranes (or membranes) were characterized in terms of hydrophilic properties and conductivity in order to confirm the successful and uniform incorporation of PANI in the membranes and evaluate the effect of PANI content on the properties of the produced membranes.

#### 3.2.1. Contact Angle

To confirm the incorporation of polyaniline in the membranes, and to track changes in the membranes hydrophobicity, as it is an important factor affecting cell attachment, the electrospun membranes were measured for their contact angle after neutralization with NaOH methanol solution (Table 2). Contact angle of the electrospun membranes before neutralization could not be measured accurately, as they are too hydrophilic and the water droplet tends to get absorbed by the surface of the membrane, altering the nanofibrous structure and finally dissolving the material. The values reported at Table 2 represent the mean value of at least six repetitions performed at different regions of each of the triplicate membranes produced under the same conditions. 

The increase of the contact angle with increasing PANI content that can be clearly seen in Table 2, can be explained by the fact that polyaniline is inherently a highly hydrophobic material, especially at its emeraldine base state, which occurs after treatment with alkali, so it inevitably enhances the hydrophobic properties of the nanofibrous membranes. The monotonic increase of the contact angle with increasing PANI content also indicates that polyaniline is uniformly incorporated in the electrospun fibres. Lastly, all four electrospun membranes fall into the category of moderately hydrophilic surfaces, as they all exhibit contact angles between 40° and 70°, which are also generally considered suitable for cell culture [46]. 

#### 3.2.2. Electrical Conductivity

The electrical conductivity of the membranes was calculated from the measured resistances with the four-point probe technique as was described in materials and methods. The Sheet Resistance (R_S_) can be obtained from the characteristic resistances *R_A_* and *R_B_* by numerically solving the Van der Pauw equation (Equation (1)).

(1) $$e^{-\pi R_{A}/R_{S}}+\;e^{-\pi R_{B}/R_{S}}=1$$

In this case, the measured resistances were: *R_A_*~*R_B_* within a mean range of 6% and not higher than 10% as is recommended in the ASTM F76, so

$$2e^{-\frac{\pi R_{A}}{R_{S}}}=1$$

$$ln2=\frac{\pi R_{A}}{R_{S}}$$

$$R_{S}=\;\frac{\pi R_{A}}{ln2}$$

The bulk resistivity is then given by the equation: $\rho =\;R_{S}d$, where *d* is the measured thickness of the conducting layer.

The latter can then be converted to conductivity by simple inversion $=\;\frac{1}{\rho }$.

**Figure 3:**
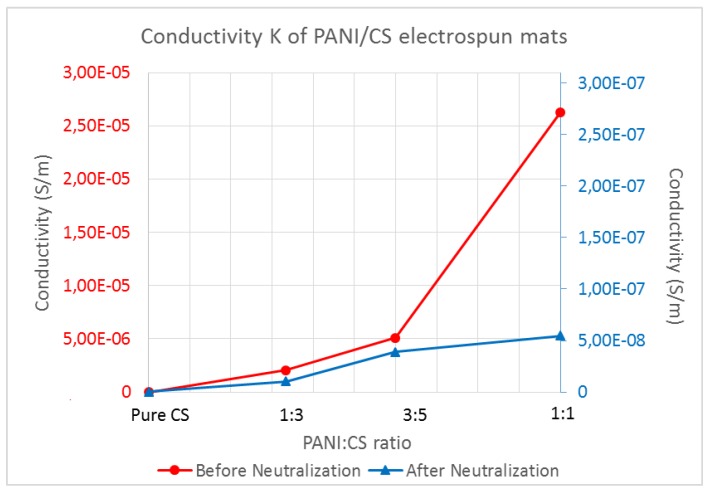
Comparison chart of membrane conductivity before and after neutralization (note the different order of magnitude for the two curves).

Applying the above, the following conductivities are shown in Figure 3.

As it can be seen from Figure 3, the dedoping that was observed visually during treatment with aqueous methanol NaOH solution, was confirmed by the conductivity measurements. A big decrease of at least 2 orders of magnitude is observed for all the samples containing polyaniline. The control pure chitosan sample did not change after the neutralization process and, as expected, it was not electrically conductive before or after the neutralization process. However, all the PANI/CS membranes, even after neutralization retained some conductivity, which was two orders of magnitude larger than the control chitosan. For tissue engineering and wound healing purposes, as only very low currents need to be applied for cell excitation, usually at the range of µA [47], the membranes with the reported conductivities are considered worth to be investigated further for their effect on cell cultures. Generally, in order for a voltage to be considered safe for electrical excitation of cells it needs to be at the range of V/cm and the generated current at the range of µΑ [47,48], therefore materials at the range of resistivity semiconductors exhibit are best candidates for this type of applications. It is also debated that even without electrical excitation, cells might be able to communicate with electrical signals they produce which can be facilitated by an electrically conducting membrane [49].

It is also observed (Figure 3) that the membrane conductivity increases with higher PANI content, as expected. There is more than one order of magnitude increase of conductivity between untreated PANI/CS 1:3 and 1:1 membrane, indicating good distribution of PANI in the membrane. When the membranes are treated with alkali, the increase in conductivity with increase of the PANI ratio is not as pronounced because the partial dedoping that occurs disrupts the charge mobility within the membrane, but still it is more than three orders of magnitude higher for the membrane with the highest amount of PANI.

### 3.3. Evaluation of Cell Attachment and Viability

To assess the cell viability, and to rule out possible and undesired acute cytotoxicity, the Live/Dead cell stain was used as a preliminary step. This test is a destructive method to assess cell viability, so it was performed on two of the human osteoblast cell seeded membranes (CH and PANI/CS 3:1). 

**Figure 4:**
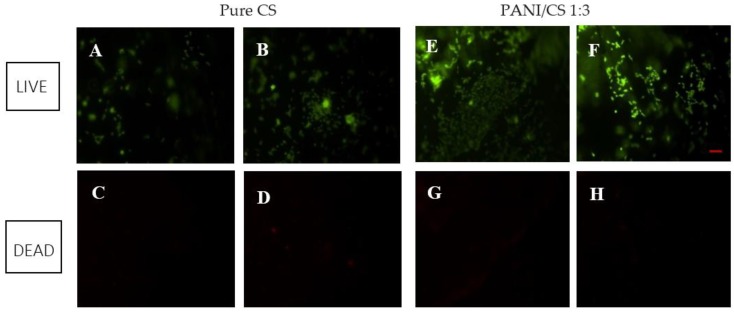
Fluorescence LIVE/DEAD stain for membranes (3 d in culture): (**A**,**B**) live at two different chitosan membrane regions, (**C**,**D**) dead staining for the same chitosan membrane regions; (**E**,**F**) live at two different PANI/CS membrane regions, (**G**,**H**) dead staining for the same PANI/CS membrane regions.

Figure 4 shows the images obtained from the fluorescence microscope for the pure chitosan membrane and the PANI/CS 1:3 membrane respectively, after staining with calcium dye which stains the live cells green (Figure 4A,B,E,F) and ethidium homodimer dye which stains red the dead cells (Figure 4C,D,G,H). From Figure 4A,B,E,F it is shown that the cells have well attached and spread on the electrospun membranes after 3 days in culture. Calcein-AM is a non-fluorescent, cell-permeant fluorescein derivative, which is converted into cell-impermeant, highly fluorescent calcein by cellular enzymes. Calcein accumulates inside live cells with intact membranes and causes them to fluorescent green. Ethidiumhomodimer-1 enters dead cells with damaged membranes and undergoes a 40-fold enhancement of fluorescence upon binding to their DNA causing the nuclei of the dead cells to fluoresce red. This double staining allows for simultaneous examination of both live and dead cells on the material surface [50]. It is evident from Figure 4, that while for green (live) fluorescence there is a high output, and a lot of cells can be seen on the membrane surface, for the same region of the membranes, there are very few to none red fluorescent spots, indicating only one or two dead cells per image (comparing Figure 4A to Figure 4C, Figure 4B to Figure 4D, etc.). It has to be noted that it was difficult to focus on the whole region of the membrane at this magnification as the membrane inevitably exhibited some wrinkles and folding in the medium.

Although this being a qualitative test, thus providing only a visual evaluation of viability, by comparing Figure 4A,B to Figure 4E,F, it is obvious that more green fluorescence per image can be seen on the blend membrane, indicating that the membrane incorporating polyaniline supports better osteoblast attachment than the control chitosan; however, no definitive conclusions should be reached yet, regarding which of the two membranes exhibits better cell compatibility. After cytotoxicity was ruled out, further quantitative tests were performed on all of the electrospun membranes containing different ratios of polyaniline and for both cell types (osteoblasts and fibroblasts).

Live and dead images were taken from the exact same spot of the membrane surface, and at the same magnification (×4), by changing filters on the microscope.

### 3.4. Cell Proliferation

**Figure 5:**
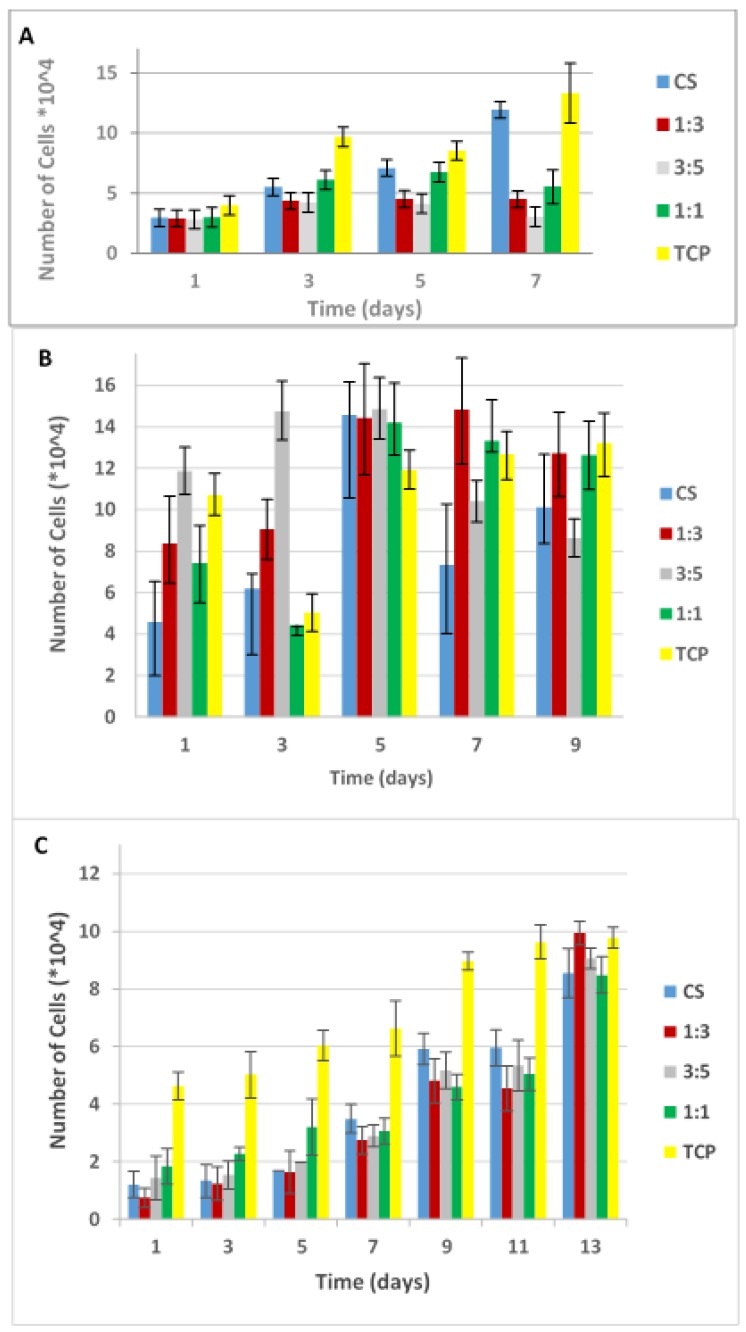
Cell numbers with time of: (**A**) osteoblasts with attachment time of one day; (**B**) osteoblasts with attachment time of three days; and (**C**) fibroblasts with attachment time of three days. All bars represent mean values from triplicate experiments and the error bars represent the ranges of the measured values.

The cell proliferation studies were performed by using the fabricated electrospun membranes of different PANI/CS ratios, and tissue culture plastic and pure chitosan as controls in order to evaluate how the added PANI affects cell growth as compared to the chitosan. Recent studies have shown that electrospun chitosan membranes enhance osteoblast and fibroblast proliferation, offering a good substrate for tissue culture. The introduction of electrical properties to the electrospun membranes is expected to have an impact on the cell behaviour, as even cells that are not considered as electroactive seem to be affected by electrical cues. The osteoblasts were allowed to proliferate for up to nine days, and the fibroblasts for up to 15 days. During preliminary experiments, it was observed that fibroblasts took longer to attach both on Tissue Culture Plastic (TCP) and electrospun membranes; therefore time 0 was defined as the 3rd day after seeding (attachment period). Over the testing period, relative cell numbers were assessed continually every other day using the Alamar Blue assay (AB). The measured fluorescence intensity was translated into cell numbers based on calibration curves (Appendix A). In Appendix A the graphs in fluorescence intensity units and indicative proliferation rates for fibroblast cells can also be found. Figure 5A–C shows the osteoblast and fibroblast cell numbers on electrospun chitosan and PANI/CS blend fibres with volume ratio of 3:1, 5:3, and 1:1, and pure CS and TCP as controls. The same three-day attachment period was applied on the osteoblast cell line too for reference.

Statistical comparison of the number of cells at the end of culture time on the different membranes and the TCP showed no significant differences (*p* > 0.05) both for osteoblasts and fibroblasts (Figure 5B,C), indicating that the nanofibrous membranes support cell proliferation in a similar way as the standard TCP. Interestingly, when the osteoblasts were left to attach for longer period (3 days instead of 1, Figure 5B) prior to the first medium change, which could cause loosely attached cells to become detached, the PANI/CS membranes, and especially the one with 3:5 PANI/CS ratio seem to promote a lot more cell proliferation during the first three days of evaluation. It is worth noting here that, for osteoblasts at Day 7, when one day was allowed for attachment (Figure 5A), the cell numbers were expected to match the cell numbers measured on Day 5, when three days were allowed for attachment (Figure 5B), as the total cell culture duration for both would be eight days. However, the cell numbers are different and this discrepancy between one and three days of attachment can be attributed to the nature of the assay that was used. More specifically, to perform the Alamar Blue assay, the supernatant is removed and new medium containing AB reagent is added to the wells. After the set incubation time, the AB containing medium is removed again and fresh medium is added, until the next time that a measurement is performed, when it is removed again. If the cells did not have enough time to reach the final phase of secure attachment on the membranes before the first measurement is taken, they may be aspirated with the culture medium. In this way, the cell density at Day 1 is reduced and, since the assay is continuous, the cells cannot proliferate normally. At this point, it is useful to take a deeper look on the way the cells adhere on surfaces. The cell attachment mechanism can be described in three phases: (A) sedimentation of cells on the substrate which is guided by electrostatic interactions; (B) integrin mediated bonding of the exoskeleton on the substrate and flattening of the cell; and (C) spreading of the cell on the cell on the substrate mediated by focal adhesions. Cell spreading seems to be accompanied by the organization of actin into microfilament bundles and the strength of adhesion becomes stronger with time [51]. Thus, going back to Figure 5A,B, while similar cell numbers are observed for the tissue culture plastic surface, when looking at the membrane data, a significant increase in cell numbers is noted: the pure chitosan shows 24% increased viability, the 1:3 membrane shows a three-fold increase, the 1:1 membrane presents a similar increase and finally the 3:5 membrane presents a massive, almost five-fold increase. These observations were evaluated statistically demonstrating *p* value = 0.032 (<0.05), thus indicating statistical significance. An asterisk has been added above the bars between which statistically significant differences have been observed. This verifies what was visually observed during the initial cytotoxicity test (Live/Dead cell stain) and was discussed in the previous section. By Day 7, all membranes containing polyaniline seem to show higher osteoblast cell numbers as compared to the control pure chitosan one (Figure 5B) and in some cases even more than the TCP, when the cells have been left to attach for three days. Rougher surfaces, such as nanofibres are usually known to provide more sites for cell attachment due to the higher surface to volume ratio. However, it is highly possible that, during the attachment period, there is a delay between Phase B and Phase C of attachment. As on Phase B the cells have not yet developed focal adhesion on the surface, when in aqueous media they may move. When this happens, the roughness of the nanofibrous surface renders it more difficult for them to reattach and spread. It has to be noted here that no difference was observed in terms of the morphology of the cells on the membranes when one day and three days of attachment are compared (Appendix B). The difference is in terms of quantity; only very few cells manage to attach on the membranes during the first day and no conclusions can be drawn in terms of morphology, as only very few scattered cells could be seen when the membranes were visualized under the SEM on the first day after seeding.

The same pattern can be found in proliferation charts of other studies too, even though this phenomenon is many times overlooked and not explained in detail. In those studies, during the first days of cell culture, TCP initially seems to outperform nanofibrous membranes, by the end of the cell culture period nanofibrous mats exhibit higher number of cells attached to them, and this can be attributed to an initial lag of the cells to reach phase C of attachment [13,28,52,53,54].

As for fibroblast proliferation, as can be seen in Figure 5C, during the first five days in culture, the membrane with the higher polyaniline content (1:1 ratio) sustains cell growth better than the control pure chitosan and the rest of the blend membranes. After Day 5, the rest of the membranes, and especially the 1:3 PANI/CS one, seem to better promote cell growth, with the latter presenting a twofold increase of cell numbers between Days 11 and 13, exceeding the value for the tissue culture plastic. The higher hydrophobicity of the 1:1 membrane may be preventing the cells to further expand on its surface. It is also again evident here that the cells take longer to attach to the membranes as compared to the tissue culture plastic, but when they do, they proliferate well and can reach viability values expressed, similar to the standard tissue culture plastic. Looking at the characteristics of these membranes, it seems that although the difference in conductivity between PANI containing membranes and the pure chitosan one is not as vast, it is very possible that in terms of cell culture this offers enough conductivity for cells to attach and proliferate better. This may also be due to the different hydrophilicity of the 1:1 mat when compared to the pure chitosan one (Table 2), but when comparing the 1:3 mat which in general showed better cell proliferation, with the pure chitosan one, the contact angle does not differ as much. Further investigation as of why the incorporation of PANI may be beneficial to cell proliferation should be conducted. These results agree with the cell proliferation results obtained by Gizdavic-Nikolaidis et al. [55], who showed an enhanced proliferation of fibroblasts on conductive nanofibrous HCl-doped 3ABAPANI–PLA mat, without any electrical stimulation. The mats with increased ABAPANI content exhibited higher contact angles (>85°), higher conductivity (>6.9 × 10^−5^ S/m) and better cell attachment and proliferation, which was significantly higher than that of glass substrate or TCP. This was attributed to the nanofibrous structure of the mats, providing more sites for attachment, as opposed to the flat surfaces of glass and TCP, however no reason was given as of why more limited fibroblast proliferation was observed for pure PLA nanofibres and blends with lower ABAPANI content as well. Considering the fact that the mats with high ABAPANI content exhibit a rather hydrophobic surface (which should be hindering fibroblast attachment), it is surprising that they perform so much better in terms of cell proliferation. This phenomenon has been studied by Jeong et al. [31] who examined the adhesion of three different types of cells (mouse skeletal muscle cells (C2C12), human dermal fibroblasts and NIH-3T3 mouse embryo fibroblasts) on PLCL scaffolds enriched with polyaniline. They found a positive relationship between PANI concentration and cell mitochondria metabolic activity and they attributed this phenomenon to either the electroconductive properties of the scaffolds or to the modified surface chemistry however excluding the surface energy, as the hydrophobicity of the membranes defined by contact angle measurements, was not significantly affected by the PANI concentration. However, in that case PLCL was used as the control, which is known for its low biocompatibility with cells and for this reason its surface chemistry is usually modified before cell culture. In the present study chitosan was used as the control which is known for its good interaction with cells. Therefore, the enhanced proliferation observed in most of the PANI containing membranes leads to the conclusion that the introduction of electroactive properties on the substrate does affect positively the metabolic activity of cells. The mechanism through which this occurs needs to be further examined and confirmed, but it is possible that these types of cells too, as it has been reported for fibroblasts in the heart [56], communicate with electrical signals through the formation of gap junction s between them, and that the electroactive surface facilitates the transmittance of these signals.

### 3.5. Cell Morphology Assessment

**Figure 6:**
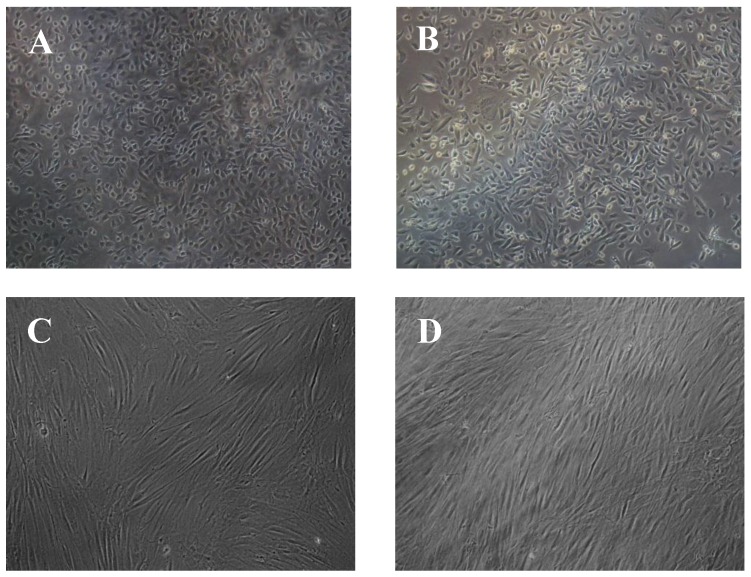
Microscope Images (10×) of: (**A**) osteoblasts on TCP; (**B**) osteoblasts on glass slides; (**C**) fibroblasts on TCP; and (**D**) fibroblasts on glass slide.

**Figure 7:**
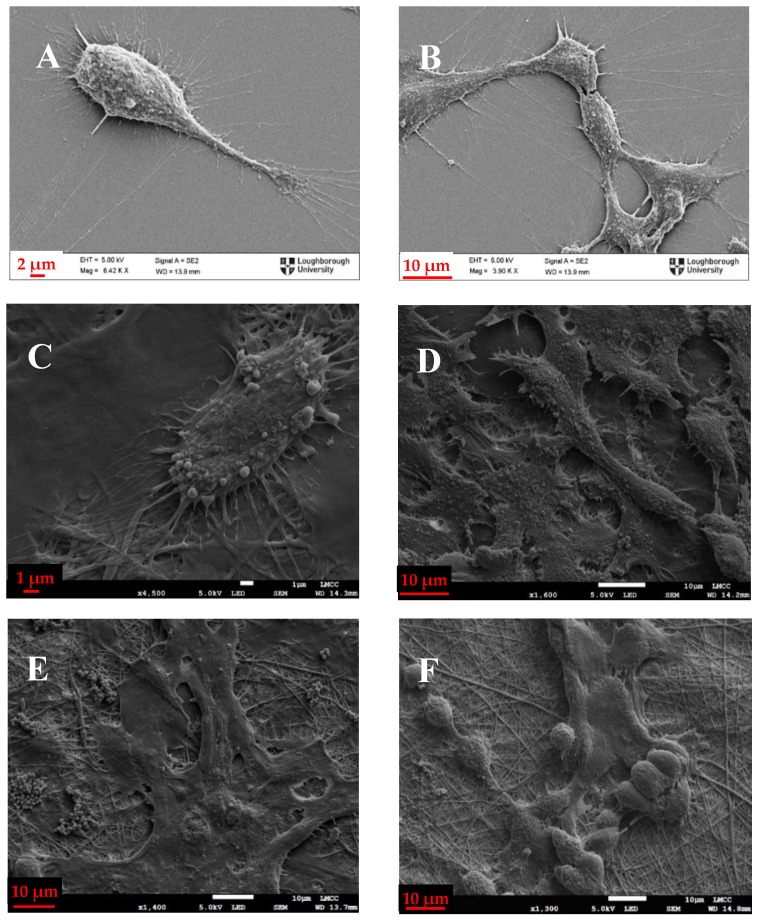
Scanning Electron Microscope Images of: (**A**,**B**) osteoblasts on glass slide; (**C**) 1:3 PANI/CS membrane; (**D**,**E**) 3:5 PANI/CS membrane; and (**F**) 1:1 PANI/CS membrane.

Cell proliferation is important, but it has to be accompanied with visual examination of the cell morphology and attachment on the membranes in order to safely draw conclusions about biocompatibility of electrospun membranes. As the electrospun membranes are not transparent, normal phase contrast microscopy that is commonly used for evaluation of cell cultures could not be employed. Therefore, scanning electron microscopy was chosen as an appropriate method to assess the morphology of osteoblasts and fibroblasts on electrospun membranes. Initially, cells were cultured on glass slides where they exhibited similar characteristics with the ones cultured on tissue culture plastic when examined using optical microscopy (Figure 6). The glass slides were then coated with Au/Pd and examined under the scanning electron microscope, to be compared with the ones on the electrospun membranes (Figure 6 and Figure 7). 

In Figure 7, it is evident that, when comparing Figure 7A,B to Figure 7C, the shape and size of osteoblasts is very similar. They exhibit flattened shape with long pseudopodia, an indication of healthy attachment and a typical size of 15–20 µm and they seem to adhere to the nanofibrous surface in the same way as they do on glass slides and tissue culture plastic (Figure 6). The same is valid for the blend membranes too (Figure 7D,E), with the exception of Figure 7F, where more globular shapes are shown, indicating that the osteoblasts were unable to spread as extensively on this membrane as on the others, possibly because of the higher hydrophobicity of this material. 

**Figure 8:**
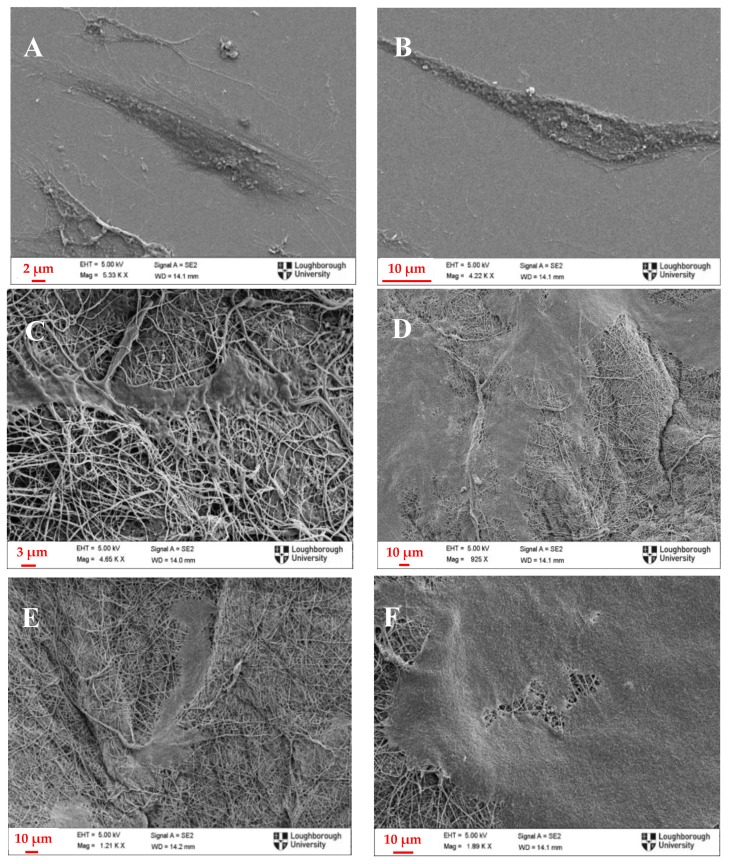
Scanning Electron Microscope Images of: (**A**,**B**) fibroblasts on glass slide; (**C**) 1:3 PANI/CS membrane; (**D**) 3:5 PANI/CS membrane; and (**E**,**F**) 1:1 PANI/CS membrane.

Similar conclusions can be drawn from Figure 8 as well, where similar flattened and elongated shapes are observed on all the surfaces, and, especially on Figure 8D,F, which were taken after the end of the culture period (13 days), it is shown that cells have successfully attached and grown all along the nanofibrous surface, almost completely covering the nanofibres. Some lumps that appear on the surface are most likely debris and pieces of dead cells which were not successfully washed away before the cell fixing treatment.

## 4. Conclusions

Novel nanofibrous membranes, which combine the benefits of conducting polyaniline and biocompatible chitosan, were produced with the electrospinning method, they were characterized in terms of hydrophilicity, diameter distribution and conductivity; and were tested for their biocompatibility with human cell lines. The nanofibrous membranes incorporating different ratios of PANI to chitosan, exhibited higher contact angle, directly related to the polyaniline content in the blend, with higher polyaniline content resulting in more hydrophobic surface. Although the neutralization process, which was necessary to waterproof the chitosan and prepare the membranes for cell culture, inevitably dedoped the contained polyaniline to some extent, it is shown that some of the conductivity was still retained. Especially regarding the mat containing 1:3 PANI/CS ratio, it seems that the retained conductivity due to PANI, together with the retained hydrophilicity due to high chitosan content showed a synergistic effect in promoting both osteoblast and fibroblast growth. None of the produced membranes showed any cytotoxicity; on the contrary cell attachment and proliferation was achieved and sustained during the culture period, even when high amounts of PANI were incorporated in the mat, contradicting observations reported in the literature when different carrier polymers are used. This is attributed to the choice of materials, which seem to exhibit a combined beneficial effect on human osteoblasts and fibroblasts.

Since the produced membranes are not cytotoxic, they are good candidates for wound healing applications, where the nanofibrous membrane would not be immersed in biological fluid and could retain fully its conducting properties. Based on these results, further research for determining how the fibroblast and osteoblast cell lines respond to electrical stimuli seems worthwhile and promising.

Another interesting finding emerging from the evaluation of cell proliferation on the substrates is that delayed adherence (1–2 days) on the nanofibrous mats is observed, as opposed to the flat tissue culture surface. From this study, it is concluded that when *in vitro* studies are performed should be considered, as it might lead to biased results regarding the biocompatibility of the tested material. However, when cells do attach, they proliferate rapidly, probably due to the facilitation of electrical signalling communication between them that the electroactive surface provides.

The conducting properties introduced by means of PANI incorporation, can also potentially offer a tool for controlled release of bioactive substances and/or electrical excitation of cells in biomedical applications.

## Figures and Tables

**Table 1:** Electrospinning parameters and resulting polyaniline–chitosan (PANI/CS) nanofibre diameters at different PANI content.

PANI/CS Ratio	Electrospinning Parameters				Nanofibre Diameter		
	Voltage (kV)	Flow Rate (mL/h)	Tip to Collector Distance (cm)	Relative Humidity (RH) (%)	Average Diameter (nm)	SD *	RSD (%) **
0 (Pure CS)	26	1	13	35	111	64	53
1:3	26	1	16	35	116	44	38
3:5	28.5	0.3	16	20	130	123	95
1:1	29.5	0.3	16	20	160	126	78

* SD: standard deviation; ** Relative Standard Deviation.

**Table 2:** Contact angle measurements for electrospun PANI/CS membranes after neutralization.

PANI/CS Ratio	Contact Angle (°)	
	Average	SD
0—Pure CS	41.3	6.72
1:3	46.67	6.47
3:5	53.09	8.17
1:1	70.34	6.35

**Figure A1:**
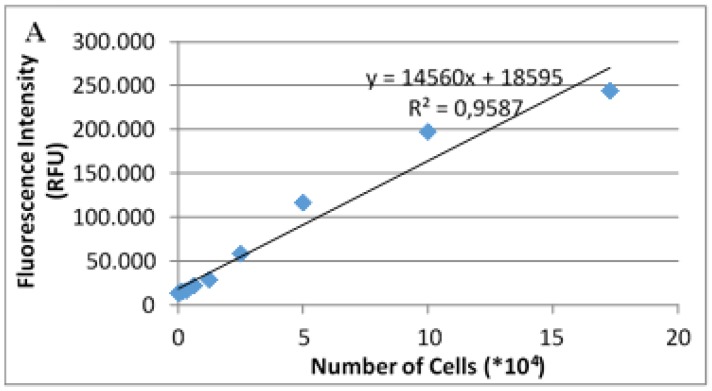

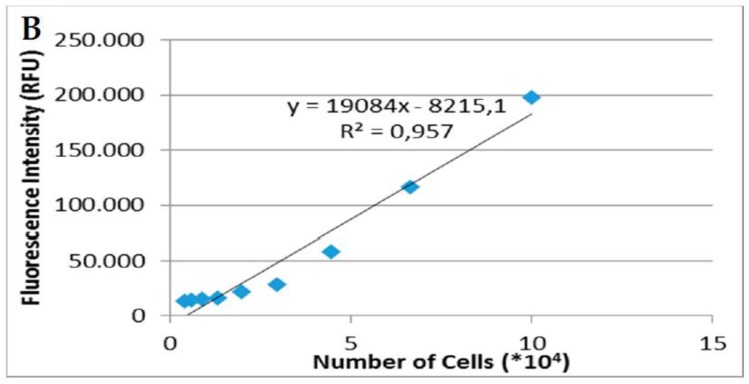
Calibration curves correlating fluorescence intensity to actual cell numbers: (**A**) osteoblasts; and (**B**) fibroblasts.

**Figure A2:**
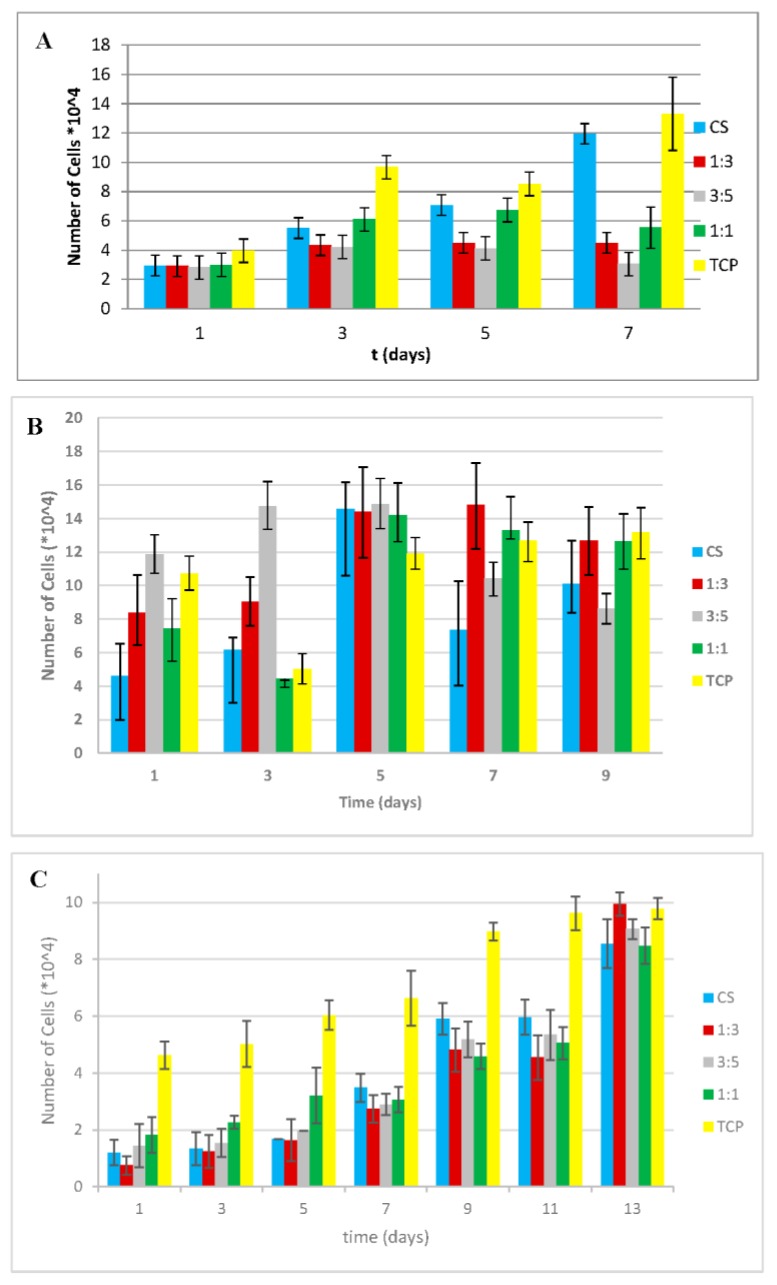
Fluorescence intensity graphs: (**A**) osteoblasts with one-day attachment time; (**B**) osteoblasts with three-day attachment time; and (**C**) fibroblasts with three-day attachment time. All bars represent mean values from triplicate experiments and the error bars represent the ranges of the measured values.

**Figure A3:**
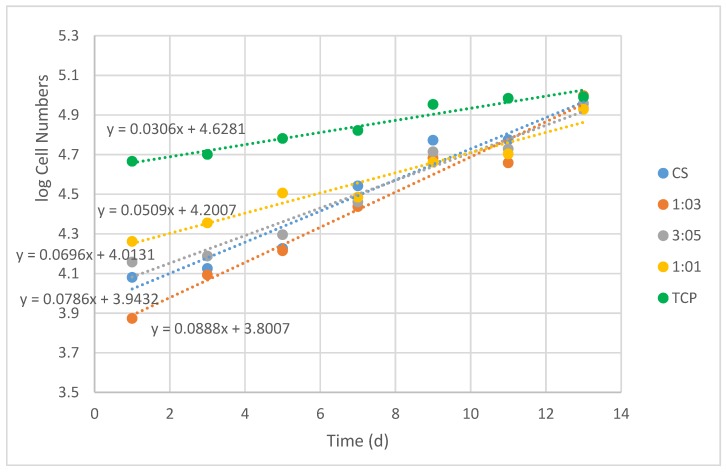
Indicative proliferation rates for human fibroblasts.

**Figure A4:**
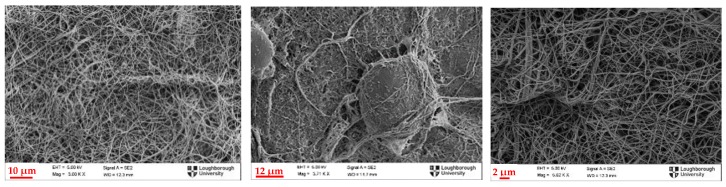
SEM images 1:1 PANI/CS membranes on day 1 after seeding with human osteoblasts.
